# An empirical investigation into people’s intention to participate in mega events tourism: applying mixture of two behavioral theoretical models

**DOI:** 10.1186/s43093-022-00175-z

**Published:** 2022-12-29

**Authors:** Yasser Tawfik Halim, Hazem Tawfik Halim, Mohamed Samy El-Deeb, Samia Adly El Sheikh

**Affiliations:** 1 October University for Modern Sciences and Arts, Giza, Egypt; 2 The British University in Egypt, Cairo, Egypt

**Keywords:** Mega events, Intention, Tourists’ trust model (TM), Deliberate actions model (DA), Observed benefits, Perspective, Observed obstacles, Interactive mechanism, Normative beliefs, Observed risk

## Abstract

The purpose of this research is to build and evaluate a theoretical model capable of forecasting public participation in mega events. This study predicts event tourism participation using a mixed behaviour model based on a trust model and a deliberate actions model. Using data from 261 local and international visitors, structural equation modelling was engaged to examine the study hypotheses. According to the study's results, observed benefits were positively connected with people’s perspective, but observed obstacles were negatively associated, and observed benefits had a positive influence on interactive mechanism and observed obstacles had a negative influence on interactive mechanism. Additionally, the study's results suggested that individuals' intention to participate in mega events was positively influenced by their observed benefits, perspective, interactive mechanism, and normative beliefs and negatively influenced by observed obstacles. Finally, we perceived that observed risk significantly moderated the associations between observed benefits, observed obstacles and perspective, observed benefits, observed obstacles and interactive, lastly, observed benefits, observed obstacles, perspective, interactive mechanism, normative beliefs and intention to participate in events. The research's outcomes have significant hypothetical and applied effects for mega-events travel.

## Introduction

Mega events are becoming a new kind of alternative tourism, with individuals travelling across the world to attend unusual events that are not available in their home country. People travel from underdeveloped to developed countries or to participate in mega events that are distinctive and unique [80]. Globalization of mega events, together with advanced technologies and facilities in host countries, in addition to enhanced communication and transportation expertise, and vacation elements all allow tourists to enjoy the local environment while attending events, have made events travel the world's fastest rising and utmost moneymaking leisure industry sector [39, 106, 124].

Guests' acceptance of events tourism has risen fast, and more global travelers are likely to travel worldwide to attend such events. Additionally, improved service standards and safety records for events services in rising nations help entice people from other countries to attend events [101].

The expanding global industry for events tourism includes both the events component and the wider financial impact of such travel [32, 141]. While experts cannot agree on a single definition of events tourism [14], some researchers feel that enjoyment and distinctiveness are two components of events tourism. Others argue that vacation components of events tourism are only "a conduit for cultural tourism" or acculturation rather than tourism [13, 19, 58, 135].

The authors of this paper assume that events tourism includes international mega events as the famous Carnivals of Mexico, Brazil and Argentina or Film Festivals as Cannes Film Festival in France, Cairo Film Festival, The Golden Globe, The Oscar as well as the opening of Cultural Museum in Egypt when the mummies were moved to the new museum. As well as the FIFA World Cup, The Champions League matches especially the final matches, the Classico between Real Madrid and Barcelona, Bull Fights and Festival of San Fermin in Spain, Saint Patrick Green Day and other. In addition to other religious and cultural mega events as Omra to Mecca and Madina and The Journey of the Holy family in Egypt.

Previous research on events tourism has mostly focused on growth of events tourism globally and on the impacts of such tourism on host destinations as [23, 60, 120, 153, 170, 171]. Numerous studies have also explored factors that impact events tourism destination selection, such as "host site cost." Vassiliadis et al. [151], "accessibility", and "Favorable currency exchange rates" as Ratten et al. [132], and "service quality of accommodation facilities" [147, 157]. The authors of this paper found that from a perspective of people's decision-making processes has yet to be studied, creating a gap in the conceptualization and modelling of tourists' behaviour connected to events travel [21, 41].

A variety of ideas explain tourist behaviour and how to persuade them [40, 138, 144]. Although one theory may sufficiently describe one component of decision-making in one setting, several theories together may better anticipate or explicate a larger variety of decision-making practices crosswise perspectives. An integrated tourist trust model (TM) Wu et al. [160] and deliberate actions model (DA) Li et al. [163, 164] and Ajzen [3] predicts factors impacting visitors' intention to participate in events tourism. This study examines combining TM and DA since both theories emphasize determining a people's behavioural intention before acting. The fact that TM and DA are extensively used to explain an extensive array of tourist-related behaviors [81, 129] justifies their integration. Due to the intricacy of visitor engagement and travel-related behaviour in events tourism, combining TM and DA may enable for more accurate prediction of both internal and external tourist behaviour. Thus, by integrating the TM and DA, this research hopes to improve and examine a theoretic model for assessing people's interest in events tourism [49, 77].

Events tourism is complex, therefore blending TM and DA may forecast internal and external visitor behaviour. Thus, merging TM and DA will enhance and evaluate a theoretical event tourism appeal model [17, 33].

This study also analyzes the impact of observed benefits and obstacles on perspectives, interactive mechanism and intention to participate in events. Aspects like interactive mechanism, perspective and normative beliefs influence people's intents to participate in events travel. Lastly, the influence of moderating of observed risk is explored. The outcomes of this research paper subsidize to the body of knowledge on leisure marketing and events tourism in particular. This research may also help service providers and events travel organizers establish an effective marketing strategy by better understanding prospective events travelers’ expectations and decision-making process.

## Literature review

### Trust model (TM)

A TM is a quantified trust representation based on trust functions. Trust can be kept on two levels: credal, where they are entertained, quantified using trust functions, and pignistic, where they are employed to make decisions, and quantified using probability functions. When choices are required, a link between the trust and probability functions is warranted. In addition to Chaulagain et al. [24], Olya et al. [117], Yoo et al. [165], and Yousaf et al. [166] have also constructed trust models [12, 121]. This article attempts to predict behaviour using certain thought outlines, which contain twofold of variables: (1) the emotional state-owned of willingness to act, and (2) the observed usefulness of a recommended action [36, 131, 149].

Harris and Dunham [71] focus on data processing to enhance fusion results in classification problems. The researchers specifically outline and implement three crucial solutions for coping with incorrect expert knowledge assumptions. It also addresses the problem of overconfidence in expert forecasts, divergent (but similar) categorization sets used by specialists to create expectations, and vagueness in expert object-to-object connections. To avoid inadequate weighting, inaccurate interpretation, and/or application of expert knowledge, these three causes of misalignment may be addressed. Deficient prior knowledge (in the Bayesian sense) may also degrade the efficiency of alignment techniques, making credal information representation (in the transferable trust model sense) an ideal arena for applying these alignment tactics. Numerous researchers have highlight the necessity to resolve unrealistic assumptions in order to improve fusion results.

### Theory of deliberate actions (DA)

The theory of logical action, (LA) Ajzen [3] presented the DA [45]. The assumption that their main referents would enjoy and approve of their activity, and that they have the vital means and probabilities to participate in the behaviour [3, 96, 97, 146]. Specifically, DA proposes that three antecedents influence an individual’s authentic implementation of an activity: perspective toward the conduct, normative beliefs, and interactive mechanism. Normative beliefs, perspectives, and interactive mechanism all affect behavioural intention [6, 48, 122, 159].

Personal views and feelings regarding a certain object are known as perspectives [6, 61, 82]. A people’s perspective is a learned habit that allows a person to react consistently to a given behaviour, product, or service [94, 163, 164]. The DA states that when someone has a positive attitude toward a behaviour, his or her intent to participate in that behaviour is similarly optimistic. "Social pressure to do or not do the desired behaviour" is a normative belief [4, 67, 148]. Normative beliefs is an individual's perception of how actions will be seen by people closest to them. DA also claims that interactive mechanism predicts goal behaviour. It refers to the observed obstacles of executing a given task [4, 104, 145]. The DA asserts that ideas of control over capitals (awareness, competence, and time) have a major effect on whether or not to participate in a behaviour.

Human behaviour may be explained and predicted using the DA model [73, 78, 86, 88, 94, 123, 130, 139, 143].

#### Hypotheses development

To generate and appraise a philosophy-centered model that empirically analyses the past history of visitors' desire to involve in events travel, this research paper incorporates the TM and DA. This study looked at the influence of observed advantages and impediments on people's views toward events tourism, as well as observed behavior control. The authors also looked at how interactive mechanism, perspective and normative beliefs affect people's intentions to attend activities. Finally, authors looked at how risk perception affected the hypothesized linkages.

### The influences of “observed benefits” and “observed obstacles” on perspective

In Behavioral Model, observed benefits are people's perceptions of an activity's rewards, as money savings [44, 93, 100]. Few studies have conceptualized observed advantages as a research concept and empirically explored their influence on people's perspectives and intentions to engage in mega events tourism. "Cost savings" "access to high-quality services", [1, 76, 108]. As well as "faster and easier events tourism" [56, 76, 63, 158] identify key drivers of events tourism participation.

Fast growth and upgrading of infrastructure and procedures have led to low pricing in developing countries. In contrast, rising pricing for events services in developed countries like the USA have increased demand for events tourism. Also, the cost gap between home and host republics has been recognized as a benefit of events tourism [14, 35, 91, 93]. In addition, events attendees may be lured to venues recognized for their high level of service and amenities [9, 28]. Finally, traits that drive events tourism may differ from those that inspire leisure tourism, with events travelers prioritizing facilities and uniqueness above geographical attributes [30, 111]. However, events tourism is a fusion of the events and tourism sectors. Peri et al. [128], and tourists go far to see events. That events tourists may combine their desired event service with a holiday may be a benefit of events tourism [55]. According to the aforementioned definition, the observed advantages paradigm includes lower prices, better event facilities, and the option to combine event service with a trip.

In spite of the fact that a behaviour is effective in reducing prospective discomfort, it may be seen as expensive, cumbersome, or unpleasant [72]. Observed difficulties influence how individuals see the activity and their motivation to participate [10, 135, 137]. Visitor barriers are observed obstacles (or constraints) in the leisure and tourist sectors [50, 74, 112]. Intrapersonal, interpersonal, and structural constraints prevent tourists from travelling for pleasure [31, 75, 79]. Intrapersonal limits include lack of interest, concern, or proficiency. Personal limitations are social links or linkages between people that occur due to the absence of others like family or friends. Inopportune amenities, pressure of time constraints, besides a deficiency of information all contribute to structural limitations [42, 118, 168].

As stated by this study, there are three major obstacles to attending large events: (i.e., intrapersonal, interpersonal, and structural). Given that mega events tourism involves visiting another country to get services and hospitality, with the potential of combining the two, it is rational to predict that mega events travelers would face related challenges as regular visitors. Interpersonal obstacles such as a lack of travel acquaintances and discontentment from friends and family might inhibit persons from travelling on the way to an overseas nation to attend mega events.

Optimistic or pessimistic perspectives toward a definite activity and a judgment of the result establish one's perspective toward the behaviour, and confidence in the benefits or obstacles causes anticipation about the outcome [57]. These subjective cost–benefit evaluations affect the perspective component of DA, which is affected by TM's observed benefit and barrier constructions [107, 150]. It has been shown that perspectives and perceptions of potential visitors to a destination are highly correlated [94, 96, 97, 150, 155, 162]. Similar interactions are expected with mega events tourism. The observed benefit level of mega events tourism may increase, while the observed obstacles level may worsen. As a result of the above debate, the subsequent hypotheses are suggested:

**H1:** Observed benefits of participating in mega events is clearly related with travelers’ perspective concerning engaging in mega events.

**H2:** Observed obstacles of participating in mega events is negatively associated with travelers’ perspective concerning engaging in mega events.

### The impact of observed benefits and observed obstacles on interactive mechanism

As previously mentioned, interactive mechanism (IM) relates to observed benefits, observed obstacles and lack of control connected with an action. Behaviour control (BC) was added to the basic philosophy of reasoned action to evaluate observed benefits and obstacles. It was included as an antecedent of behavioural intention in response to concerns that the philosophy of purpose act assumed absolute volitional control over behaviour and hence neglected observed restrictions on people's abilities to accomplish desired actions [2, 7, 16, 20]. It is critical to study behaviour, such as people's perspectives on software piracy as a two-tailed example, and the predictive behaviour of each aspect. For instance, interactive mechanism was shown to be a significant component in the choice to cheat on an exam or shoplift, but observed benefits perspective was found to be more significant in the decision to utilise information technology. While it has been shown that observed benefits perspectives have a role in unlawful software copying behaviour, the impact of interactive mechanism has received little attention. It is necessary to do research comparing the efficiency of interactive mechanism, the observed benefits perspective and the observed obstacles in predicting ethical and unethical behavior [127].

According to Lestari et al. [98], Ajzen and Driver [5], and Dun et al. [37], BC are favorably connected to the quantity of resources a person feels he or she owns either positively to sorts of benefits he or she gains or negatively related to the observed number of obstacles. Previous DA study showed that trust of control over external resources to influence desire to participate in an activity. Ohme [116] said that interactive mechanism might be used to highlight the adoption of a new technology as an example, to include participants in balancing the positive and negative elements. Prior study has focused on the observed benefits connected with the acceptance of an innovative technology. However, the weighting of contradicting variables is seen as critical in explaining a stimulus's possible acceptability [8, 27, 136]. BC is operationalized more widely than observed obstacles, affecting not just intentions but also actual behaviour [7, 15, 110]. In order to identify the real benefits or constraints that support or prohibit people from participating in the behaviour, a greater knowledge of the notion BC is necessary via investigation of its antecedents. When it comes to mega events tourism, a range of benefits as well as constraints may support or limit potential visitors' capacity to govern their behaviour. For example, benefits of attending a unique event happened once, and exposing to different culture, or deficiency of awareness, scheduling restrictions, or deficiency of a travel mate might alter people's interactive mechanism with events tourism. Therefore, we looked at observed benefits and obstacles as a BC antecedent. Constructed from the above debate, the subsequent hypotheses are advanced:

**H3:** Observed benefits of participating in mega events is certainly accompanying with travelers’ interactive mechanism.

**H4:** Observed obstacles of participating in mega events is negatively associated with travelers’ interactive mechanism.

### The impacts of observed benefits and observed obstacles on intention to participate in events

Zhang et al. [169] discussed customers' intentions for green product dissemination. They observed that consumers' observed benefits influenced their intentions to purchase energy-efficient items. The researchers surveyed 1025 British consumers regarding Smart Home Technologies (e.g. smart metering) and discovered that customers avoided utilising the technology owing due to the observed obstacles of privacy leakage, which has a negative effect on purchase intention. Observed benefits and obstacles connected with the distribution of energy-efficient items are often intrinsically tied to practise and will have a direct impact on residents' energy routines. However, there is a dearth of particular studies on the effect of observed consequences on energy-efficient adoption.

Bordia et al. [18] concluded that the intention to share information is more likely to occur when the customer perception results in favourable consequences such as incentives, increased reputation, or even job advancement. Additionally, the researchers discovered that users of virtual communities of practise were more motivated to engage and exchange information if they observed that doing so aided in their establishment as experts. In other words, the "market" is made up of buyers and sellers who trade information, products, and services in return for observed benefits and gained value. The higher the observed value or benefits of sharing certain information, the more probable it is that a person would share it. Additionally, the social exchange framework tells us about the negative impact of observed obstacles associated with the intention to sharing information. Obstacles perception is a sort of transaction cost that manifests as observed impediments to doing an activity. If the observed value of an exchange outweighs observed obstacles, the predicted negative impacts of evaluative cost on intention for information sharing may be overcome. In other words, when observed benefits are low, the negative link between observed obstacles and the intention to share information may be larger than when observed benefits are high.

Therefore, from the previous discussion we can set forth the following hypotheses.

**H5:** Observed benefits are positively related with tourist intent to participate in events.

**H6:** Observed obstacles are negatively associated with tourist intent to participate in events.

### The impacts of normative beliefs, perspective, and interactive mechanism on intention to participate in events

Interactive mechanism and good normative beliefs are associated with a favorable perspective toward an activity. A number of experiential researches have looked at the relationship among the three DA categories and tourist travel intents [29, 125]. Similar interactions might be predicted in the area of major events tourism. People may engage in events tourism if they have favorable feelings about it and believe the outcome will be satisfactory. Similarly, since participating in mega events tourism is costly, the opinions of others (e.g., family and friends) may influence participation intentions. To be more specific, those contemplating mega event tourism may expect assistance from family and friends. Being able to attend mega events influences people's behaviour. People's motivation to engage in events tourism decreases if they lack resources (time, money) and information (about events tourism).

While not widely examined in the context of mega events tourism, earlier research has revealed the beneficial impacts of normative beliefs, perspective, and interactive mechanism on behavioural intent to involve in mega events travel. They observed that all three DA components were considerably associated with people's behavioural intention to participate in events travel, with perspective being the biggest forecaster, shadowed by normative beliefs and interactive mechanism. Furthermore, Han et al. [69] and Liang et al. [103] revealed that normative beliefs, perspective and interactive mechanism significantly affected visitors' travel intention. Following the aforementioned rationale, the following ideas are proposed:

**H7:** Perspective toward mega events is positively linked with tourist intent to participate in events.

**H8:** Interactive mechanism of mega events is positively related with tourist intent to participate in events.

**H9:** Normative beliefs of mega events are positively allied with tourist intent to join in in events.

### Observed risk

China spread COVID-19 worldwide in early 2020, leading numerous countries to limit travel, socialising, and other activities. Tourism and hospitality lost customers and laid off jobs. The second wave devastated several countries that controlled the pandemic. Travel restrictions were too permissive, resulting in more tough restrictions restricting tourism-related enterprises. After vaccination and reducing restrictions, most travellers avoid going out [156].

Deliberate actions (DA) theory is effective for studying human behaviour and decision-making. Recent studies examined COVID-19-related tourist behaviour. Han et al. [68] studied how COVID-19 knowledge affects attitude and subjective norm and how observed risk moderates causal links between several aspects. Investigated pandemic travel intent.

Despite COVID-19 experiments, risk is rarely used in decision-making and tourist prediction research. According to multiple research, health risk and other types of risks assessment and perception, together with interacting mechanisms, strongly influence travel decisions. Like Moon [109] and Liu et al. [105], they advocated adding elements with causal linkages and moderators to the classic theory of deliberate actions (DA).

The literature describes the use of events services as a complex phenomenon driven by many elements (e.g., accessibility, cost, and personal views), with observed risk being a prominent factor in explaining service use [95]. Observed risk in the context of TM relates to people views about the anxiety, tension, discomfort, and dread associated with being a tourist [11, 43, 140]. Observed risk is positively related to people's willingness to travel and attend foreign events, but not in TM context. Observed risk was shown to be a key influence in people's choice to contemplate attending international events tourism by Yang et al. [163, 164], Richards & King [134]. Observed risk may act as a moderator for the associations described in this research, in addition to direct correlations. Few studies on the moderating influence of observed risk has been done, to the authors' knowledge. Intention to engage in mega events tourism will likely vary depending on observed risk and observed advantages and obstacles. Casidy and Wymer [22] pinpointed that observed risk influences service sector customer behavior. Also, Social media reviews may increase risk perceptions, especially in the service industry. Even if a service provider has a good reputation, a few unfavorable reviews can deter potential clients. Many studies examine how observed risk moderates the relationship between several variables like satisfaction, loyalty, perception, intention, and decision making. According to the literature, four types of observed risks—financial, social, performance, and psychological—considered as the moderator constructs. Consequently, the following hypothesis is proposed:

**H10:** Observed risk of tourists positively moderates the relationship between

**(H10a)** Observed benefits and perspective**,**

**(H10b**) Observed obstacles and perspective,

**(H10c)** Observed benefits and interactive mechanism,

**(H10d)** Observed obstacles and interactive mechanism,

**(H10e)** Observed benefits and intention to participate in mega events**,**

**(H10f)** Observed obstacles and intention to participate in mega events**,**

**(H10g)** Perspective and intention to participate in mega events**,**

**(H10h)** Interactive mechanism and intention to participate in mega events**,**

**(H10i)** Normative beliefs and intention to participate in mega events**.**

**Fig. 1:**
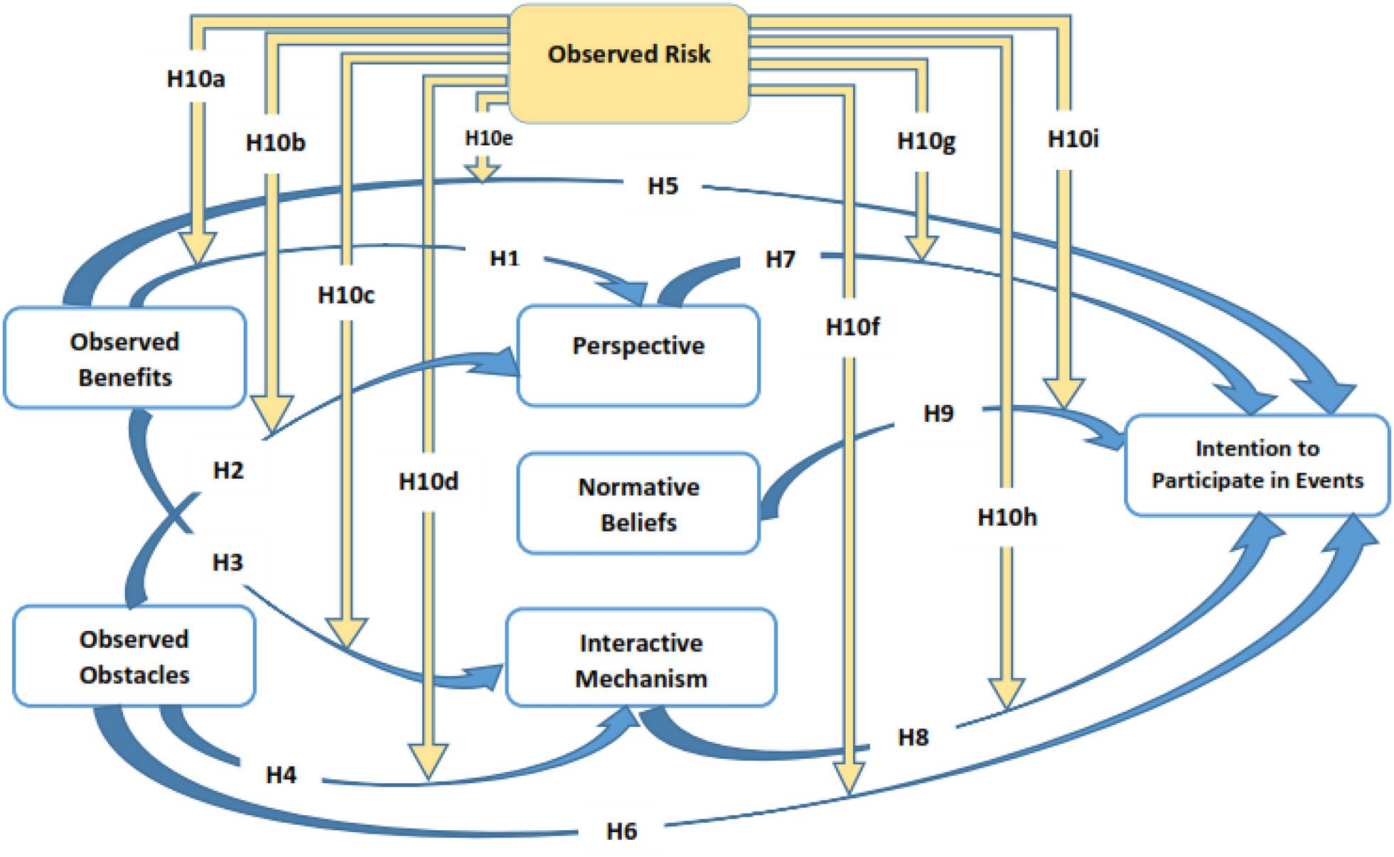
Model of the research

The theoretical context in Fig. 1 briefly exemplifies Hypotheses from 1 to 10.

## Methodology

### Survey instrument

The data collection tool was adopted and adapted from previous literature to further ensure their validity and reliability and thus were derived from prior studies. The observed obstacles were assessed using 12 items adapted from [46, 62, 85, 98, 115, 119]. The three-item scale used to measure perspective was adjusted from [4, 6, 38, 59, 102]. Three questions were amended from [99, 100, 152, 162], to assess the normative beliefs. Three items evaluating interactive mechanism were borrowed from Patwary et al. [126],Wu and Chen [161], and three items indicating behavioural intention were adapted from [34, 89, 90]. Only observed benefits were not derived from prior studies. Observed risk was quantified using a single question in which respondents indicated the severity of the danger (i.e., the major circumstance that impacts them the most). Observed risk was quantified using a five-point scale (i.e., 1 = not risky, 2 = mild risky, 3 = moderately risky, 4 = risky, and 5 = very risky).

### Sampling and data collection

Domestic and international travelers on vacation who are interested in travelling and may attend a mega event in a foreign country were the study's target group. The research paper used a self-administered questionnaire to gather data. Visitors were randomly handed questionnaires. The authors employed convenience sampling. Also, two filtering questions were used to guarantee that only qualifying responses were included in the sample of the study at hand and these questions were: Are you interested in attending mega events? Are you interested in the idea of visiting a mega event in a foreign country? The data collection date was from April to November 2021. There were 261 responses. The data was collected from actual events tourists attending the ceremony of the transfer of "Royal Egyptian Mummies Paraded" from the Egyptian Museum in Tahrir to the National Museum of Egyptian Civilization in Fustat, Cairo, Egypt, on April 3, 2021, and the opening of Pharaonic Rams-Road (Sphinx avenue) at Karnak Temple in Luxor, Egypt, on November 25, 2021. The sample was drawn from the population using a nonprobability sampling approach known as convenience sampling. For the major demographics of the sample (see Table 1 for the whole sample profile), showed around 53% males and 38% aged 35–54. Approximately 66% of applicants had a BSc. degree, and 28% had a household income of $2500 > $3500. Parley, 52% of participants were married, and 57% were domestic guests.

**Table 1:** Sample outline

Demographic features	*N*	Percentage
*Gender*		
♂	137	52.5
♀	124	47.5
Total	261	100
*Age*		
18 > 25	32	12.3
25 > 35	53	20.3
35 > 45	44	16.9
45 > 55	56	21.5
55 > 65	43	16.5
65 > 75	31	11.9
Over 75	2	0.8
Total	261	100
*Education*		
High school	48	18.4
BSc	172	65.9
MSc	34	13
PhD	7	2.7
Total	261	100.0
*Monthly income*		
Less than 500 $	55	21.1
500 > 1500	63	24.1
1500 > 2500	46	17.6
2500 > 3500	73	28
3500 > 5000	18	6.9
5000 and more	6	2.3
Total	261	100
*Marital status*		
Married	136	52.1
Divorced	37	14.2
Single	79	30.3
Widowed	9	3.4
Total	261	100.0
*Tourists*		
Domestics	148	56.7
International	113	43.3
Total	261	100.0

**Table 2:** Results of the measurement model

Constructs	Standardized loadings	Construct reliability	AVE
Observed benefits		0.72	0.52
1. If I travel to an overseas country to attend a mega event, I will be excited for the new experience	0.71		
2. If I travel to an overseas country to attend a mega event, I will enjoy a higher level of hospitality at a much lower cost than my country	0.76		
3. If I travel to an overseas country to attend a mega event, I will have the chance to combine my desired event amenities and service with a trip	0.72		
4. If I travel to an overseas country to attend a mega event, I will expose to advanced technology during the event compared to the technology of my country	0.77		
Interpersonal obstacles		0.91	0.77
1. I have no one to travel with me (have no travel companion) if I travel to an overseas country for attending an event	0.89		
2. My family and/or supports are not concerned in traveling with me to a foreign country to attend an event	0.86		
3. I feel uncomfortable because of cultural variations if I travel to an overseas country to attend an event	0.92		
4. I have difficulty of verbal communications if I travel to an overseas country to attend an event	0.86		
Structural obstacles		0.81	0.59
1. I do not have time to travel to an overseas country to attend an event	0.80		
2. I do not know much about the events in foreign countries	0.81		
3. Family and/or job commitments would keep me from traveling to a foreign country to attend an event	0.82		
Intrapersonal obstacles		0.88	0.72
1. I have concerns about my personal security if I travel to a foreign country to attend a mega event	0.78		
2. I feel frightening because of absence of control over the external environment if I travel to a foreign country to attend a mega event	0.91		
3. I am doubtable of the healthful behaviors from the foreign country if I travel to an overseas country to attend a mega event	0.85		
Perspective		0.89	0.72
1. Traveling to an overseas country to go to an event would be a good idea	0.81		
2. I like the idea of traveling to an overseas country to attend a mega event	0.86		
3. Traveling to an overseas country to go to an event would be a pleasant experience	0.87		
Normative beliefs		0.87	0.71
1. Persons who inspire my activities consider that I would travel to an overseas country to go to an event	0.91		
2. I would travel to an overseas country to go to an event because many of my friends have already traveled abroad to attend events	0.82		
3. Persons who are important to me consider that I would travel to a foreign country to attend an event	0.82		
Interactive mechanism		0.85	0.67
1. Traveling to an overseas country to go to a mega event would be entirely within my control	0.81		
2. I would be able to travel to an overseas country to attend a mega event	0.82		
3. I have the capitals, information, and aptitude to travel to a foreign country to attend a mega event	0.83		
Intention to participate in events		0.93	0.87
1. I predict that I should travel to an overseas country to attend an event in the near future	0.92		
2. I organize to travel to a foreign country to attend a mega event in the near future	0.94		
3. I intend to travel to an overseas country to go to an event in the near future	0.88		

*AVE* average variance extracted

**Table 3:** Matrix of discriminant validity

	1	2	3	4	5	6	7	8
1. Perspective	**0.838**							
2. Interpersonal obstacles	0.266	**0.867**						
3. Intention to participate in events	0.654	0.179	**0.917**					
4. Normative beliefs	0.544	0.088	0.615	**0.843**				
5. Interactive mechanism	0.731	0.322	0.642	0.478	**0.818**			
6. Observed benefits	0.541	0.075	0.533	0.550	0.433	**0.632**		
7. Structural obstacles	0.194	0.539	0.125	0.049	0.325	0.125	**0.753**	
8. Intrapersonal obstacles	0.324	0.711	0.234	0.141	0.322	0.085	0.419	**0.868**

*AVE* average variance extracted

The off-diagonal elements are the inter construct correlations, and the diagonal elements (in bold), the squared root of AVEs

### Data analysis

Prior to analysis, the data were normalised. Histograms and box plots indicated a normal distribution of data. All skewness and kurtosis measurements were within 2 standard deviation range [51–54]. The tolerance values for all constructions were more than 0.2, indicating that multicollinearity was not an issue. The researchers followed [154] in applying the EFA and CFA before conducting the SEM analysis [154].

For the purpose of discovering the underlying components of observed hurdles and benefits, an exploratory factor analysis (EFA) was used. The entire measurement model was examined using first-order confirmatory factor analysis. EFA and DA were included as part of the CFA (i.e. observed behavioral, perspective, control and normative beliefs). Second-order constructs were viewed as impediments [26, 155]. As a result, observed obstacles were viewed as a second-order construct. Structural equation modelling was used to evaluate the conceptual model and research hypotheses (SEM) [53].

Finally, AMOS was utilized to do a multi-group moderation test on the structural model (pairwise comparison). Multi-group models evaluate structural models in multiple groups [84, 142]. It can estimate within-group parameters including loadings, routes, and correlations. Chi-square and fit indices may be calculated for each group and for the multigroup model. Multigroup predictive studies must first determine if a measure has equal qualities across groups to avoid confounding substantive group differences with measurement attributes. The data set was segmented by a category variable and separate models are estimated for each section. Multi-group comparisons are performed to test if the model's predicted relationships change based on the moderator's value. The probable relationships between the characteristics were examined using multi-group moderation to compare low (*n* = 48), moderate (*n* = 88), and high (*n* = 125) observed risk groups.

## Results

### Exploratory factor analysis

The underlying features of the observed obstacles and observed benefits constructs were determined using an exploratory factor analysis. The data were mined by means of major axis factoring and varimax spin. The Kaiser–Meyer–Olkin test, which measures sample adequacy (MSA), was greater than 0.5 (MSA = 0.864), and the correlation matrix's overall significance was less than = 0.001, with a Bartlett test of sphericity value of 1981.230. Items with factor loadings equal to or greater than 0.5 were included in the data analysis. Variables with eigenvalues equal to or greater than one were also deemed significant. Due to factor loadings of less than 0.5, one intrapersonal item (my inclusive positive perspective is an anxiety when moving to a distant nation to attend a mega event) and one operational obstacle piece (weather would be a most important issue if I traveled to a foreign country to attend a mega event) were excluded from the data analysis. The EFA found a four-factor model that explained seventy-three percent of the variance. Interpersonal hurdles accounted for parley forty percent of the variance, intrapersonal obstacles for parley sixteen percent of the variance, structural impediments for about ten percent of the variance, and observed benefits for 7.3 percent of the variance. Cronbach's alpha was used to assess the reliability of the measurement scales. The alpha coefficients of the accessible grading systems fluctuated from 0.71 to 0.93. When compared to the appropriate point of reference value (0.70), the scales are deemed credible. They might be put to good use in future studies [114].

### Measurement model

Before evaluating the subsequent-order dimension model and examining the structural model, the full former-order measurement model was examined. In the CFA analysis, EFA (intrapersonal, interpersonal, and structural barriers, as well as observed benefits) and DA components were employed (interactive mechanism, normative beliefs, perspective, and intention to participate in events). The model fit was acceptable, according to a significant chi-square statistic (chi-square = 442.383, *df* = 270) and other fit indices such as the root mean square error of approximation (RMSEA = 0.048), comparative fit index (CFI = 0.95), goodness-of-fit index GFI (90), normed fit index (NFI = 0.92), and incremental fit index (IFI = 0.95) [65]. The composite reliability (CR) technique was used to assess the measurement scales' dependability. All dimensions in Table 2 had CR values greater than the planned value of 0.8, indicating that they were dependable [47, 114].

The authors looked at the convergent validity of the AVE values to determine the average variance recovered. All eight components had AVE values above than the suggested threshold of 0.5, indicating that they had strong convergent validity, as shown in Table 3 [47]. In order to evaluate the discriminant validity, square roots of AVEs were related to the correlation between variables. There was excellent discriminant validity, as evidenced by AVE values with square roots exceeding the correlations between two components. Cronbach's alpha values, average extracted variance (AVE), and composite reliability (CR) measurements for each group have been presented. All factor loadings were statistically significant and above.5, and Cronbach's alpha values were above the required level. 70 [113].

Second-order factor analysis indicated that the model fit was strong, as evidenced by a substantial chi-square value (chi-square = 461.730, *df* = 281) and other fit indices (RMSEA = 0.052, IFI = 0.95, NFI = 0.91, CFI = 0.97, and GFI = 0.91). A substantial convergent and discriminant validity was also proven by the second-order measurement model (Table 4). It was revealed that interpersonal hurdles had the biggest influence on the observed obstacles constructs, followed by intra-personal and structural difficulties (Table 4).

**Table 4:** Results of the second-order measurement model

Constructs	Standardized loadings	Construct reliability	AVE
Observed obstacles		0.89	0.62
Interpersonal obstacles	0.91		
Structural obstacles	0.67		
Intrapersonal obstacles	0.82		

### Analysis of the structural model

**Fig. 2:**
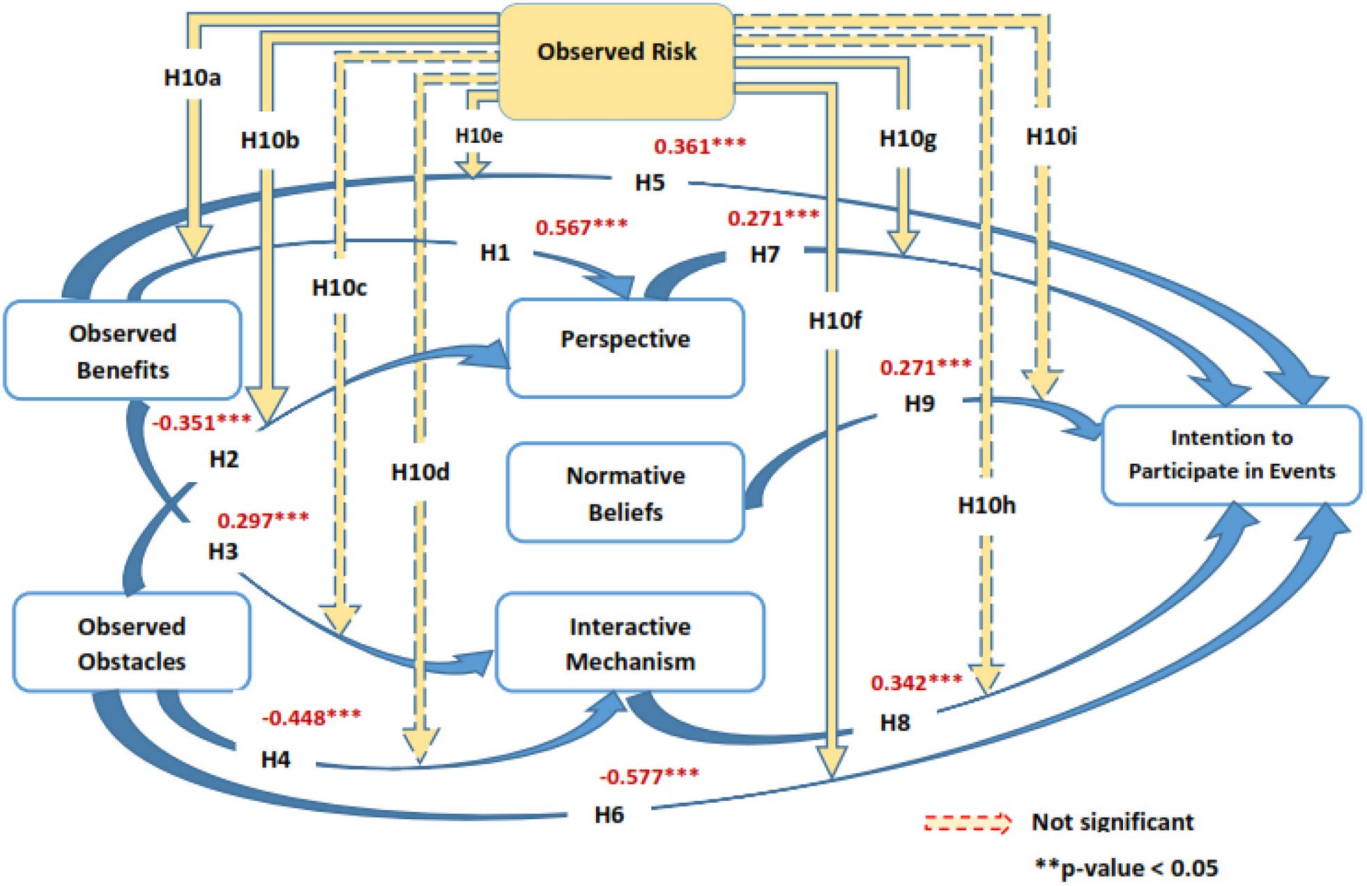
Structural modeling analysis results

The study's hypotheses were tested using SEM. There were no issues with the model's fit. 2.1 was below the cutoff value of 3 for the chi-square to degrees of freedom ratio. RMSEA is 0.066, GFI is 0.91, CFI is 0.91, NFI is 0.91, and IFI is 0.92 [65] and [66]. According to the findings, hypotheses 1 through 9 were proven to be significant (Fig. 2, Table 5). Observed benefits (hypothesis 5), Perspective (hypothesis 7), interactive mechanism (hypothesis 8) and normative beliefs (hypothesis 9) were favourably correlated with the intention to engage in mega events, while observed benefits were negatively correlated with observed obstacles (hypothesis 6).

**Table 5:** Results of testing hypotheses (moderation hypotheses are excluded)

Structural paths	Standardized path coefficients	Hypothesis supported (yes/no)
Hypothesis 1: Observed benefits → Perspective	0.567***	Yes
Hypothesis 2: Observed obstacles → Perspective	− 0.351***	Yes
Hypothesis 3: Observed benefits → Interactive mechanism	0.297***	Yes
Hypothesis 4: Observed obstacles → Interactive mechanism	− 0.448***	Yes
Hypothesis 5: Observed benefits → Intention to participate in events	0.361***	Yes
Hypothesis 6: Observed obstacles → Intention to participate in events	− 0.577***	Yes
Hypothesis 7: Perspective → Intention to participate in events	0.271***	Yes
Hypothesis 8: Interactive mechanism → Intention to participate in events	0.342***	Yes
Hypothesis 9: Normative beliefs → Intention to participate in events	0.270***	Yes

***p < 0.01; **p < 0.05; *p < 0.10

There is some evidence to support hypothesis 10 that one's perspective toward participating in mega event (hypothesis 10 g) is moderated by one's observed risk, which lends credence to hypotheses 10a, b, e, f, and g. We found strong positive and negative impacts on perspective for both the high-risk and low-risk groups, based on our findings (Table 6).

**Table 6:** The moderating effect of observed risk

	ObBen → Persp	ObObs → Persp	ObBen → IMec	ObObs → IMec	ObBen → Int	ObObs → Int	Persp → Int	IMec → Int	NormB → Int
Low risk	0.872	− 0.421	− 0.219	0.514	0.854	− 0.511	0.273	0.319	0.329
Moderate risk	0.881	− 0.334	− 0.313	0.349	0.761	− 0.315	0.430	0.363	0.433
*z*-score	0.048	0.834	0.315	− 0.874	0.045	0.722	0.567	0.355	0.345
Low risk	0.239	− 0.225	− 0.229	0.847	0.229	− 0.224	0.227	0.328	0.353
High risk	0.866	− 0.423	− 0.446	0.872	0.766	− 0.332	0.527	0.466	0.632
*z*-score	4.470***	2.231**	0.754	0.323	3.310***	3.131**	2.729***	0.854	1.471
Moderate risk	0.881	− 0.345	− 0.263	0.263	0.762	− 0.245	0.430	0.363	0.218
High risk	0.827	− 0.234	− 0.414	0.255	0.635	− 0.234	0.255	0.446	0.353
*z*-score	− 0.328	1.345	0.320	− 0.045	− 0.227	1.345	− 0.844	0.520	1.125

*ObBen* Observed Benefits, *ObObs* Observed Obstacles, *Persp* Perspective, *IMec* Interactive Mechanism, *NormB* Normative Beliefs, *Int* Intention

***p < 0.01; **p < 0.05; *p < 0.10

## Discussion

By combining the TM with the DA, this research established and evaluated a theoretical model for examining people' intentions to participate in mega events tourism. The study's findings on hypotheses 1 and 2 suggested that observed benefits were favorably correlated with people's perspectives toward mega events tourism, whereas observed obstacles were adversely associated. Consistent with previous research, our findings indicate that both the obstacles and benefits constructs have a significant role in shaping people's perceptions of mega events tourism. Prior research, however, produced contradictory conclusions about the magnitude of the influence obstacles and benefits have on people' perspectives and behavioural intentions (e.g., [155]. According to the outcomes, benefits like experience, hospitality and lower cost than own country and other benefits (hypothesis 1: Beta = 0.567) ensured a bigger impact on perspective than obstacles like security and health issues (hypothesis 2: Beta = − 0.351) on the participants' outlook. This suggests that people's perceptions of the positive aspects of mega event tourism as a new life time experience, better hospitality with lower cost than one’s own country, combining the mega event with a trip benefit and exposure to more advanced technology are more important than their perceptions of the negative aspects as health hazards, or like not having travel companion, cultural variations and verbal communication. Additionally, this research discovered a major reason for the detrimental impacts of observed obstacles on interactive mechanism that matches with [169].

In support of hypothesis 3, it was shown that people' observed benefits are substantially related to their control of and availability of capital (funds), availability of information, and aptitude to travel to a foreign country to attend a mega event when traveling to a distant country (hypothesis 3: Beta = 0.297).

Research demonstrated a negative connection between observed obstacles and interactive mechanism such as a control of capitals, information, and aptitude to travel to a foreign country to attend a mega event (hypothesis 4: Beta = − 0.448) in support of hypothesis 4. Wang, Deng, and Petrick [155] found that travel behaviour is negatively influenced by observed obstacles like not having travel companion, cultural variations and difficulties of verbal communication as well as health hazards and other external fears. Additionally, this research discovered a major reason for the detrimental impacts of observed obstacles on interactive mechanism that matches with [169]. Additionally, this research discovered a major reason for the detrimental impacts of obstacles on interactive mechanism, namely that people with high obstacles perceive that flying to a distant country to attend a mega event is out of their control.

This could be explained that if one part of the independent variable which is “observed obstacles” in this case (H4) then the dependent variable “interactive mechanism” will be negatively impacted by 0.448 part, assuming all other factors constant which is a major negative impact.

Research confirmed a positive connection between observed benefits and desire to participate in mega events by having the intention to organize a travel to mega events (hypothesis 5: Beta = 0.361) in support of hypothesis 5, this finding corroborates a recent research by [25]. This could be explained that if one part of the independent variable which is “observed benefits” in this case (H5) then the dependent variable “intention to participate in Mega Events” will be positively impacted by 0.361 part, assuming all other factors constant.

Research demonstrated a negative connection between observed obstacles and tourists’ desire to participate in mega events tourism by predicting when and where to travel (hypothesis 6: Beta = − 0.577) in agreement with Kim [87, 89], a which support of hypothesis 6.

In support of hypothesis 7, it was shown that people perspectives like a pleasant experience of the idea of travelling are substantially related with their desire to participate in mega events tourism (hypothesis 7: Beta = 0.271). This finding corroborates a recent research by Kim et al. [87, 89], which found that perspective is a favourable predictor of intention. More precisely, the findings indicate that people's favourable perspectives will have a beneficial influence on their intent to participate in mega events travel.

Normative beliefs like the inspiration of family and friends was shown to be a significant interpreter of intent to participate in events (hypothesis 9: Beta = 0.270). In agreement with prior research results. The findings of Hagger et al. [64], Harb et al. [70] demonstrate that people’s intention are influenced by the ideas of others and tend to act in line with their expectations. In support of hypothesis 8, the study's findings indicated that participants' interactive mechanism was positively connected with their intention to participate in mega events travel (hypothesis 8: Beta = 0.342). Indeed, perceptions of interactive mechanism such as aptitude and availability of capital had the highest impact on intent to participate in activities, followed by perspective and normative beliefs. These findings suggest that when people think they lack the requisite means and knowledge for mega events tourism and believe that going to a foreign nation to attend a mega event is out of their control, they likely to avoid events tourism. Researchers like Reason [133], Lee et al. (2012) and Jiang et al. [83] have all failed to find substantial links between interactive mechanism and intention in earlier events tourism studies. Hypothesis 10 was partially supported by the study's findings, which showed that observed risk (Low, Moderate and High) functions as a moderator between the constructs. According to the findings, those who believe their trip involves a high level of danger have a bigger impact on their perspective and intention to engage in events than those who believe it entails a lower risk.

Moreover, the findings of the research under study are consistent with previous studies as mentioned which enhances and supports the research findings.

We can conclude that hypotheses from 1 to 9 were accepted and supported while the tenth hypothesis H10 (a, b, e, f, g) were significantly supported but H10 (c, d, h, i) were insignificant and not supported.

### Theoretical implications

Destination marketing and the subject of events tourism both benefit greatly from the study's findings, which are substantial theoretical contributions. As previously stated, this study used both the TM and DA to determine participants' plans to participate in events tourism. We looked at how the DA components relate to the observed benefits and challenges instead of immediately linking them to behavioural intention. As far as the authors’ awareness, this research provides a complete theoretical foundation for understanding how people's behavioural intentions for events tourism develop by contextualising and include the TM and DA in a prediction model. Mega events tourism research can be improved by combining the TM and DA models, which account for 47% of the variation in people's intents to participate in mega events tourism.

As previously stated, observed obstacles have not been explored in order to ascertain people's perspectives and intentions to participate in mega events tourism. According to the results of this research, observed obstacles were a major predictor of perspective. Thus, this study established the applicability of obstacles in mega events tourism, demonstrating that they are a critical and viable concept in events tourism research. Additionally, observed obstacles predicted a large and reasonably high percentage (27%) of the BC construct, and the BC had the highest influence on intent to participate in events, afterwards the normative beliefs components of DA. As discussed earlier, BC was included into the inventive philosophy of rational act in order to account for observed obstacles and restrictions that limit people's willingness to conduct desirable behaviours. The study's findings indicate that associating BC only with external facilitating or impeding variables, as previously recommended in DA research, may be insufficient for understanding the true obstacle or restriction reasons that prohibit people from engaging in the activity. As a result of the current study's results, it is thought that including observed obstacles into DA may lead to an enhanced accepting of the idea of BC and its backgrounds.

To conclude, this is the first study to add and assess the effect of observed risk on the frequency of events tourism. The outcomes of this study reveal that the application of the observed risk construct is widened as a moderator construct that interrelates with the previous factors that impact people's willingness to take part in mega events.

### Managerial implications

There are major practical implications for decision makers and marketers, event amenities and technology providers, as well as event travel service providers like events tourism, travel agencies, even if the research offers considerable theoretical contributions. A more inclusive and operational marketing strategy can be developed and implemented to foster a more favourable perception of events tourism among possible travelers by identifying the factors that influence people's engagement with mega events tourism and the associated parties in the events tourism industry. Study results suggest that event tourism facilitators should educate visitors about the benefits of event tourism. Host country facilitators of events tourism are responsible for promoting their own country's events offerings to visitors. Digital marketing and social media should be used to spread the word about the various facilities' good service records, reputations, specialties, and accreditations. The lower cost and competitive advantage of technical upgrades to event operations should also be communicated to potential attendees (in comparison to other competing nations). Workshops and seminars aimed at informing and educating potential event tourists about the host country's high-quality event services, as well as their availability and lower costs and simplicity of booking, should be held. Research shows that an event vacation is a substantial side benefit of tourism, despite the fact that many visitors travel for pleasure and excitement. Visitors to the host country for the event should be informed and educated about the possibility of vacationing while there.

According to the study's findings, perspectives and BC were adversely connected with observed obstacles and mega event visitor involvement ambitions. For these reasons, it is vital to help potential events tourists overcome the hurdles that prohibit them from participating in events tourism. In terms of intrapersonal obstacles, tactics geared at minimizing observed risk, security issues, and anxiety may encourage tourists to come to a foreign nation to attend a significant event. Events leisure industry organizers would inform and enlighten possible vacationers about events tourism using reliable and credible information to assist overcome structural impediments. Promotional activities (e.g., discounted airfare and accommodations, promotion of host country tourism attractions) should be communicated by event tourism facilitators to raise awareness and generate interest among potential event tourists' family and friends with the intention of mitigate the undesirable effect of interpersonal obstacles. It is critical for events tourism organizers to apply an inclusive marketing approach that targets not only potential events travelers, but also their immediate surrounds, including family and friends, in order to understand the value of subjective criteria in the decision-making process. To do this, promotional and informational materials should be distributed to friends of potential tourists and, relatives allowing them to join in the decision-making process and improve their knowledge of events tourism. Mega event tourism has a strong desire to participate by tourists, and the risk level connected with these circumstances modifies the relationships between observed benefits, perspective, and interactive mechanism. Event tourism locations must announce the factors that influence people's decision to participate in event tourism in order to effectively manage their destinations. Results from this study can help planners and event organisers to understand the expectations and thought processes of potential mega-event attendees. In light of this data, event service providers and destination managers can devise effective marketing strategies to boost client information acquisition and highlight product differentiation in order to keep a competitive advantage.

### Limitations and future research

The present work makes significant theoretical and practical advances to the field of mega events tourism. However, this study has significant limitations, and further research is necessary to corroborate the study's conclusions. The survey gathered data from Egyptian residents and foreign visitors to Egypt. Future study that gathers data from different countries may provide useful information for examining psychographic variations in the behavioural intentions of events tourists.

TM and DA were successful in forecasting visitors' desire to participate in events travel, but upcoming research may evaluate the effects of other antecedents on people's intent to participate in events tourism using alternative theoretical frameworks. When it comes to events visitors, further research could examine the impact of additional factors such as the cost and availability of various tourist packages. It also didn't consider the specific qualities or prior experiences of the respondents. Follow-up studies that examine people's behaviour intents in relation to their unique characteristics such as frequency of international travel, demographic characteristics and prior events tourism experience such as age, marital status, gender, level of education and income may provide additional insight in this regard. Because of the above-mentioned factors, future research could have a completer and more holistic picture.

## Conclusion

Finally, event tourism is a fusion of the events and tourism sectors [128], in which tourists go far to see such Mega events. Mega events tourists may combine their desired event service with a holiday [55]. As such, Mega events tourism is not exactly the same as ordinary, leisure tourism in which people or tourists travel only to enjoy a holiday with their family or friends, in other words Mega events tourism include the pleasure of tourism in addition to a desire to attend the event in question, for example a group of friends travelling across the world from Brazil their home country to Qatar to attend the matches of their national team in the upcoming FIFA World Cup in 2022. It is understandable that they would want to enjoy their trip as tourists and require all amenities and services as other tourists but their main aim is to attend the matches of their national team and had it not been for this event, they would not have taken the risk of their journey. Attending the matches will involve an added risk of the event which is attending the matches or attending Carnivals or Film festivals or other, depending on the event in question. As such Mega event tourism involves additional risk other than ordinary tourism risk. This has motivated the authors of this paper to adopt a mixed theoretical approach thus adding a theory to complement the most commonly used theory by previous researchers in tourism literature to explain and predict a tourist’s intention to engage in a specific behavior at a specific time and a particular place, which is the behavior required by marketers ‘the visiting behavior’, and this theory is the Theory of Deliberate Acton (DA) or the Theory of Reasoned Action [45] which was extended to the Theory of Planned Behaviour [4] as a model to predict individual’s intention to engage in a behavior over which people have the ability to exert self-control and mixing it with the tourist Trust Model (TM).

From the above discussion, and in an attempt by the authors of this paper to generate and appraise a model to empirically investigate tourists’ intentions to attend Mega events, the authors of this paper have resorted to incorporate the Theory of Deliberate Actions (DA) components-variables (interactive mechanism-normative beliefs-perspective-intention to participate in Mega Events) with the Trust Model (TM) components variables (emotional willingness to act and observed usefulness i.e. observed benefits versus observed obstacles) in order to look at how added observed risk moderates the hypothesized linkages between the constructs of the independent variables with the intention to participate in the mega events (the dependent construct). This is the added value and main contribution of this paper. The authors added how the DA components relate to the observed benefits and challenges instead of only and immediately linking them to behavioural intention as other previous articles have done. As far as the authors’ awareness, this research provides a complete theoretical foundation for understanding how people's behavioural intentions for events tourism develop by contextualising and include the TM and DA in a prediction model. The findings indicate that Mega events tourism research can be improved by combining the TM and DA models, which account for a considerable 47% of the variation in people's intents to participate in mega events tourism.

Thus by combining the TM with the DA, this research established and evaluated a theoretical model for examining people' intentions to participate in mega events tourism, which is the main addition of this paper to theoretical literature of Mega Events Tourism.

To conclude, this study attempts to add and assess the effect of observed risk on the frequency of events tourism. The outcomes of this study reveal that the application of the observed risk functions as a moderator that interrelates with the factors that impact people's willingness to take part in mega events. Thus a tourist's perspective toward participating in mega event is moderated by his/her observed risk. The results indicate strong positive and negative impacts on perspective for both the high-risk and low-risk groups, based on the findings of this paper. Thus observed risk (Low, Moderate and High) functions as a moderator between the constructs. Moreover, according to the findings, those who believe their trip involves a high level of danger have a bigger impact on their perspective and intention to engage in events than those who believe it entails a lower risk.

The research offers a view of a model capable of forecasting public participation in mega events that gives any country a snapshot of the market when designing a mega event, similarly like the opening of the Egyptian Grand Museum in the near future.

## Data Availability

The authors declare they have full access to all study data, take fully responsibility for the accuracy of the data analysis, and have authority over manuscript preparation and decisions to submit the manuscript for publication.

## Abbreviations

AVE: Average Variance Extracted
BC: Behaviour Control
BSc.: Bachelors of Science
CFI: Comparative Fit Index
CR: Composite Reliability
DA: Deliberate Actions
EFA: Exploratory Factor Analysis
GFI: Goodness-of-Fit Index
IFI: Incremental Fit Index
IM: Interactive Mechanism
IMec: Interactive Mechanism
Int: Intention
LA: Logical Action
MSA: Measures Sample Adequacy
MSc.: Masters of Science
NFI: Normed Fit Index
NormB: Normative Beliefs
ObBen: Observed Benefits
ObObs: Observed Obstacles
Persp: Perspective
PhD: Doctor of Philosophy
RMSEA: Root Mean Square Error of Approximation
SEM: Structural Equation Modelling
TM: Trust Model
♀: Female
♂: Male
